# Kaposiform Hemangioendothelioma in Adolescent-Onset Scoliosis: A Case Report and Review of Literature

**DOI:** 10.1155/2020/1839053

**Published:** 2020-02-07

**Authors:** Junho Kim, Yeon Ho Kim, Hyoungmin Kim, Bong-Soon Chang, Choon-Ki Lee, Sam Yeol Chang

**Affiliations:** Department of Orthopaedic Surgery, Seoul National University College of Medicine & Seoul National University Hospital, Seoul, Republic of Korea

## Abstract

*Background and Objectives.* Kaposiform hemangioendothelioma (KHE) is a vascular tumor of very low incidence, which occurs mostly in children and infants. The tumor is recognized for its locally aggressive, yet rarely metastatic behavior. It may cause consumptive coagulopathy known as Kasabach-Merritt phenomenon. We report a distinctive case, where an 11-year-old boy is presented with progressive thoracolumbar scoliosis without any symptom or neurological sign. *Case Report.* The patient underwent spinal deformity correction via posterior pedicle screw instrumentation and fusion, along with tumor biopsy. The pathology report confirmed KHE. The patient did not show a prominent progression of scoliosis after the surgery without any further treatments. *Conclusions*. Many of scoliotic patients do not have any apparent cause, thereby regarded as idiopathic scoliosis. The presented case is where kaposiform hemangioendothelioma is likely to be linked to the patient's scoliosis. We demonstrate the possibility of secondary scoliosis should always be kept in mind of orthopaedic doctors. We also conclude that secondary scoliosis does not show exacerbation after growth completion.

## 1. Introduction

Kaposiform hemangioendothelioma (KHE) is a rare tumor of vascular origin, which usually presents in childhood [1]. KHE is located most commonly in the trunk, extremities, retroperitoneum, and to a lesser extent, head and neck and long bones [2]. The tumor is known to be of borderline nature. Complications are common, causing severe morbidities [3]. Kasabach-Merritt phenomenon (KMP), consumptive coagulopathy triggered by abnormal growth of capillary endothelial cells, is also known to be linked to KHE [4]. We present here a case of an 11-year-old boy, at first thought to have had adolescent idiopathic scoliosis, but later diagnosed with KHE of T11/T12 spine.

## 2. Case Report

**Figure 1:**
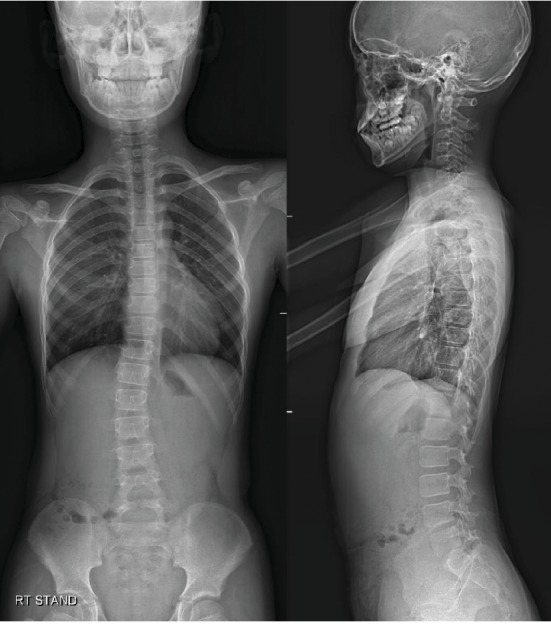
Whole spine plain radiograph. AP and lateral view at the initial visit, at July 9^th^, 2012.

**Figure 2:**
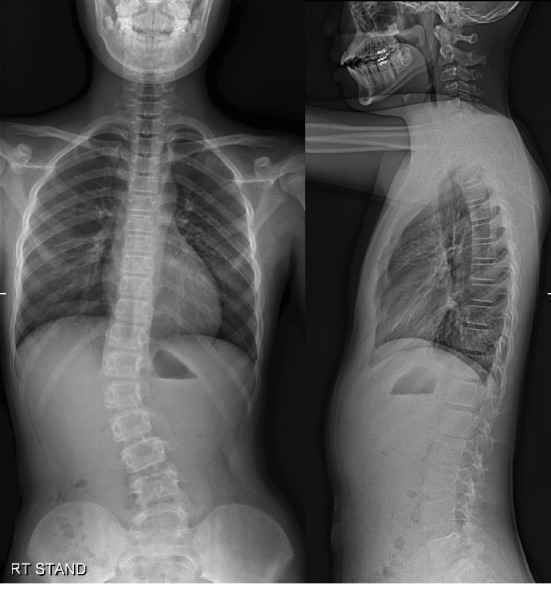
Whole spine plain radiograph. AP and lateral view when brace therapy was started, at June 16^th^, 2014.

A boy with mild scoliosis has attended the outpatient clinic for the first time. He had no medical history of being treated for other diseases and no particular genetic predisposition. He was 11 years and 10 months of age at the first visit. A thoracolumbar curve convex to the right side was identified. Cobb's angle was measured 14.8 degrees, and Risser stage 0 was confirmed via whole spine plain radiograph (Figure 1). He was regularly followed up with whole spine plain radiograph every 6 months. At 13 years and 9 months of age, Cobb's angle of the scoliotic curve had progressed to 30.1 degrees. Since he was still evaluated to be at Risser stage 0, brace therapy was applied (Figure 2).

**Figure 3:**
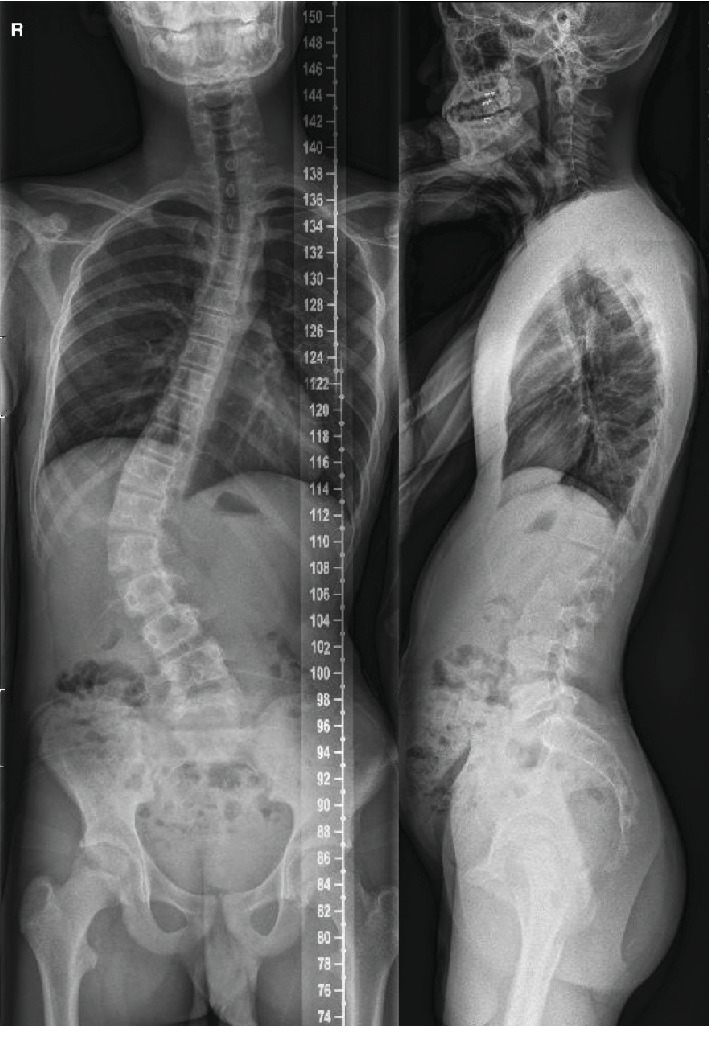
Whole spine plain radiograph. AP and lateral view when surgery was decided for the patient, at December 28^th^, 2015.

At the age of 15 years and 3 months (Risser stage 3), Cobb's angle had worsened despite the brace therapy he had received for 18 months. He had neither pain nor neurological signs. The only symptom is that he felt the back was a bit curved; however, he still managed to participate in sports activity without much trouble. Laboratory evaluation at the time of hospital admittance included CBC, electrolyte panel, infection panel (CRP and ESR), and coagulation labs, which were all within normal range. His right thoracic hump was assessed 9.0 cm from the midline, and 31 degree of hump inclination was measured. In the whole spine simple radiograph, the scoliotic curve apex was at L2, with end vertebrae at T8 and L4. Cobb's angle of 52.0 degrees was recognized, which decreased to 35.7 degrees on the bending film (Figure 3).

**Figure 4:**
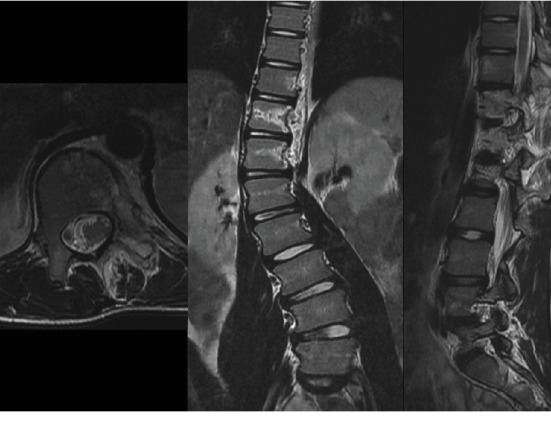
MRI scan at the time of surgical intervention decision. (a) Axial view of T11 vertebral body level. Left side of vertebral body, pedicle, and paravertebral musculature are involved with the tumor. (b) Coronal view. The tumor is located on the left sides of T10–T12 vertebrae, while T11 vertebral body is the most affected by the tumor. (c) Sagittal view. The tumor is dispersed among T11 and T12 vertebral bodies, passing the pedicles, extending to the laminae.

**Figure 5:**
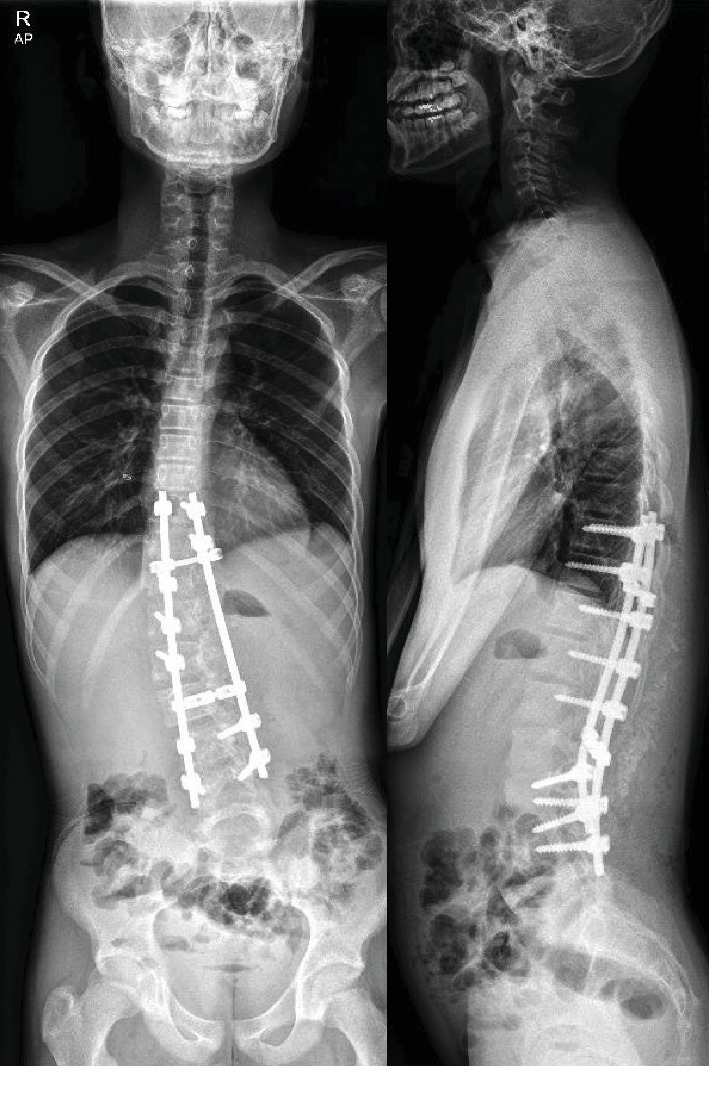
Whole spine plain radiograph. AP and lateral view at the immediate post-op, January 2^nd^, 2016.

Because the scoliosis had progressed despite the patient had approached to growth termination, MRI scan was taken. It showed high-signal intensity lesion from the left side of T11 and T12 vertebral bodies, along the left pedicles to laminae on T1-weighted images (Figure 4). Hypoplasia of left transverse processes and left ribs at T11~L1 levels were noted also, resulting in scoliosis at T-L junction. The radiologists had described the lesion to be benign vascular malformation such as angiomatosis. Since the scoliosis was progressing despite brace treatment, deformity correction surgery was planned along with an open biopsy for the tumorous lesion. During operation, specimens for both frozen and permanent biopsy were obtained from left pedicles of T11 and T12. Intraoperative frozen pathology result showed only fat tissue without sign of tumor cells. No obvious inflammation or tumor appearance could be noted on the posterior spinal structure, and therefore, the deformity correction using posterior instrumentation and fusion through T9 to L4 was done (Figure 5). No further removal of tumorous lesion was performed.

**Figure 6:**
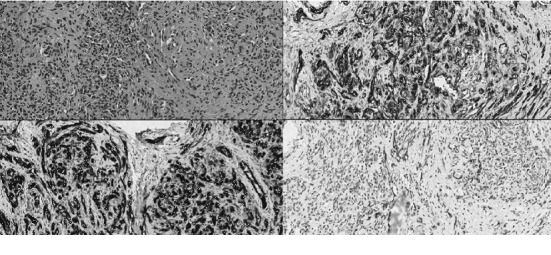
Pathologic slides of the tumor tissue obtained during the surgery (×40 high power fields). (a) HE staining. (b) Slide showing CD31 positive. (c) Showing CD34 positive. (d) Showing D2-40 positive.

The official pathology result, however, revealed KHE. The pathology slide shows infiltrating nodules with compact spindle cells surrounded by slit-like luminae (Figure 6). The immunohistochemistry staining showed negative for GLUT-1, positive for CD31 and CD34, and focal positive for D2-40, which is consistent with KHE. The patient was discussed with the department of oncology about the need for additional excision surgery for KHE. The conclusive decision was to follow-up yearly with MRI scan, without going through another surgery.

**Figure 7:**
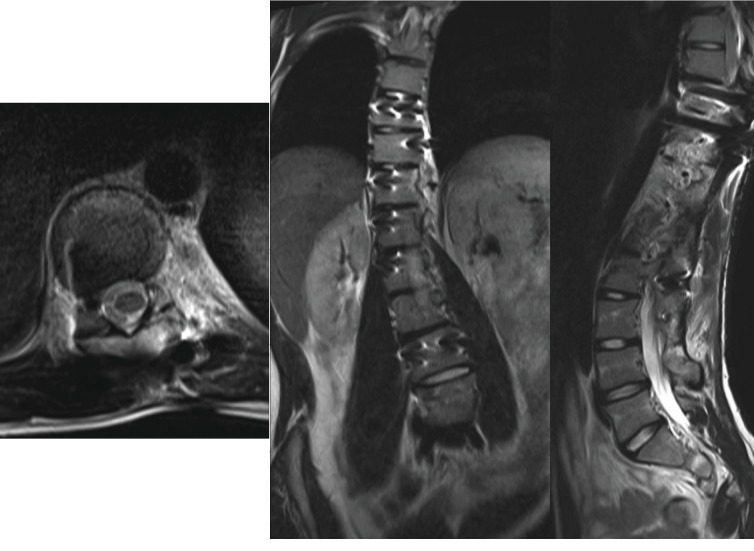
MRI scan at the follow-up visit to outpatient clinic, at July 30^th^, 2018, 2 years and 7 months are passed since the surgery. No tumor growth was detected on the follow-up scan. (a) Axial view at the T11 vertebral body level. The tumor is still confined to pedicle and left paravertebral muscular area. (b) Coronal view. The tumor is still located along the left sides of T10–T12 vertebrae. (c) Sagittal view.

**Figure 8:**
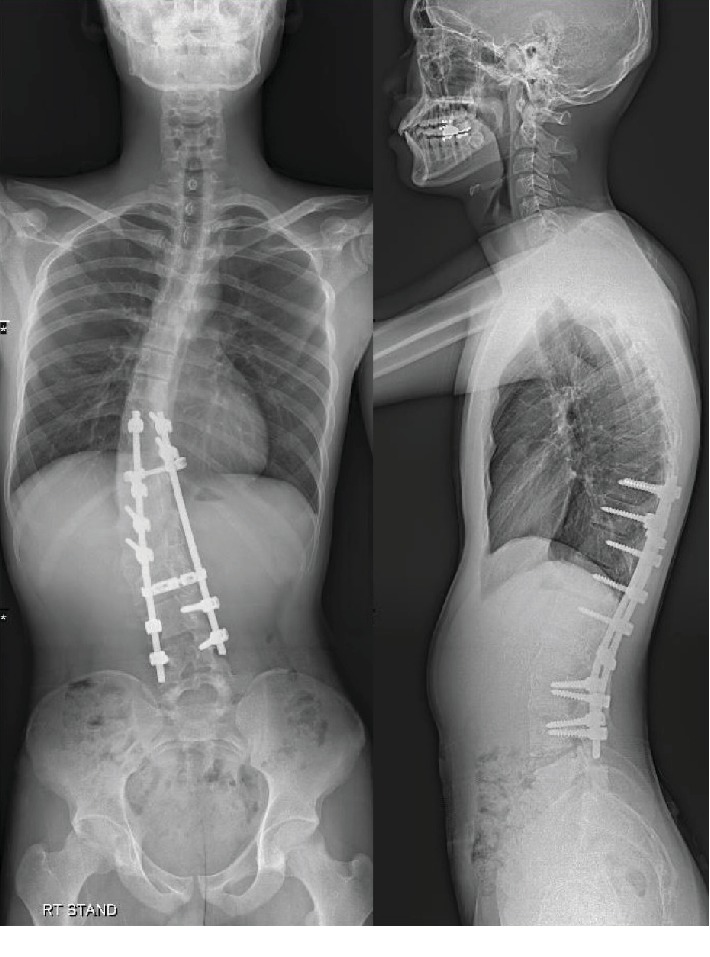
Whole spine plain radiograph at the follow-up visit to outpatient clinic, at March 11^th^, 2019, 3 years and 4 months are passed since the surgery.

After a follow-up for more than 3 years with an annual MRI examination, KHE showed equivocal progression (Figure 7). Cobb's angle of thoracic scoliosis progressed minimally from 25 degrees (at immediate post-op) to 31 degrees at the post-op 3 years and 3 months of follow-up (Figure 8).

## 3. Discussion

KHE is known to be a tumor of locally aggressive character. Patients are mostly presented with KHE early in childhood; about half of the incidences occur at birth [1, 5]. It rarely metastasizes distantly [6] but can show invasive pattern to the surrounding tissue, thus producing an ill-defined margin of the mass [5]. Reddish skin lesion and consumptive coagulopathy are the hallmarks of KHE [7]. The current case was challenging to diagnose, since the patient had no notable skin lesion and other signs or symptoms. The preoperative laboratory tests did not present clues to coagulopathy. Only with the tissues obtained intraoperatively could we conclude the KHE diagnosis.

The tumor is well recognized for not going into spontaneous regression, unlike infantile hemangioma [1]. No consensus has been reached about optimal treatment for KHE. Due to very low incidence of KHE, prospective studies had not been conducted [8]. Schmid et al. insisted that meticulous surgical excision must be considered as the first line of treatment for KHE, while Ji et al. proposed that sirolimus could be regarded as the first option [4, 9]. KHE often manifests as an immovable enlarging mass [10]. Our patient's lesion was located deep inside, alongside the thoracic vertebral bodies. The authors had waged benefits of additional excision surgery over potential morbidity. Below are the reasons that were considered primarily and decided not to conduct further surgery.

First, the severity of complications regarding KHE relies on the age of onset. About 90% of all KHE incidences rise before the age of 10 [1]. Children usually have a tumor greater than 5 cm in diameter, while adult-onset KHE tumors are smaller [5, 6]. Two-thirds of the adult patients have a tumor with a diameter less than 2 cm. Adult-onset KHE does not show thrombocytopenia, whereas over 50% of children with KHE show KMP [1, 5, 6, 11]. KHE occurring in adults also seem to be sensitive to chemotherapy [8, 11, 12]. The patient was already over 15 years old when the confirmative pathologic diagnosis of KHE was made and had not shown any symptoms or signs in accordance with KHE. Therefore, we expected the upcoming clinical course of the KHE to be dormant. The authors agreed to treat the patient for KHE only when he showed symptoms in the upcoming future, because every therapy has side effects, and the patient does not have enough gains through it.

Second, the instrumentation and fusion without tumor removal would be sufficient to suppress the coiling effect of the KHE, if any. Zhu and his colleagues had reported a case of KHE in a 14-year-old girl, in where the KHE was located on the convex side, spanning 9 levels of vertebrae including the apex of her scoliotic curve [13]. The girl did not show progression of the curve after 3 years, who received instrumentation of scoliotic vertebrae only without tumor removal. We authors had expected the patient's scoliosis to not show exacerbation, as we performed instrumentation and fusion on encompassing the vertebral levels affected by the KHE.

Third, another surgery might stimulate KHE in dormant state [14]. It is practically impossible to resect KHE thoroughly, so the additional surgery may flare up KHE activity, rather than removing the tumor entirely. Therefore, the authors had decided not to conduct further surgical means. It is possible that the first surgery might have triggered quiescent KHE of the patient. So the importance of regular surveillance to check for activation of KHE must not be overlooked. In conclusion, close follow-up with routine MRI scans was chosen for the patient instead of undergoing distressful tumor removal, which may lead into unfortunate tumor reactivation.

Idiopathic scoliosis is the most common cause of spinal deformity among adolescents. It should always be kept in mind to weigh the rate of scoliosis progression and remnant growth of the patient altogether. If they do not match, then suspicion to the cause of the scoliosis should be raised, followed by tests to identify the culprit.
